# Singapore Programme for Integrated Care for the Elderly (SPICE)—an integrated model of care to enable frail elderly to be cared for in the community

**Published:** 2012-09-04

**Authors:** Ho Chun Keong, Wong Loong Mun, Leo Feng, Josephine Huang, Jason Cheah

**Affiliations:** Agency for Integrated Care (AIC), Singapore; Agency for Integrated Care (AIC), Singapore; Agency for Integrated Care (AIC), Singapore; Agency for Integrated Care (AIC), Singapore; Agency for Integrated Care (AIC), Singapore

**Keywords:** SPICE, elderly, Singapore, Integrated Care, individualised care plan, PACE

## Abstract

**Purpose:**

Alternative models of care for the frail elderly are needed in Singapore in view of the rapidly ageing population, increased longevity and increase in the complexity of elderly care needs. The objective of the programme (SPICE) is to delay and avoid institutional care for the frail elderly by meeting their complex care needs in the community.

**Theory:**

SPICE is modeled after the US-based Programme for All-Inclusive Care of the Elderly (PACE), which delivers comprehensive care for NH eligible individuals. Evidence has shown that the community-based, integrated and comprehensive programme decreases overall hospital re-admission, average length of stay in hospital and visits to the emergency department (ED), decreases caregiver stress and improves overall satisfaction with care arrangements [1, 2].

**Method:**

In SPICE, an inter-disciplinary team (IDT) delivers an integrated suite of medical, nursing, rehabilitation, social and personal care services, both in the centre and at the participant’s home, dependent on the needs. A comprehensive assessment, regular re-evaluation and detailed individualised care plan (ICP) is tailored and implemented to enable these frail elderly to avoid institutional care.

**Results:**

37 participants have been recruited for the programme since October 2010. Preliminary 6-month pre-post analysis showed that caregiver stress decreased by 42% and participants perception of their own health increased by 18%. The total number of hospital admissions also decreased by 66.6%, average length of stay in the hospital decreased by 47.8% and the number of visits to the ED decreased by 50% over the 6 months.

**Conclusion:**

The results suggest that SPICE is effective in enabling the frail elderly to reduce and avoid institutional care and improving their overall satisfaction with care arrangements. Further investigations with matched controls are needed to study if the long-term results.

Powerpoint presentation available at http://www.integratedcare.org at congresses – San Marino – programme.
